# Changes in Left Ventricular Function and Outcomes After Trancatheter Edge-to-Edge Repair for Secondary Mitral Regurgitation

**DOI:** 10.1016/j.jscai.2024.101345

**Published:** 2024-02-23

**Authors:** Stamatios Lerakis, Annapoorna S. Kini, Gennaro Giustino, Malcolm Anastasius, Federico M. Asch, Neil J. Weissman, Paul A. Grayburn, Saibal Kar, D. Scott Lim, Jacob M. Mishell, Brian K. Whisenant, Michael J. Rinaldi, Samir R. Kapadia, Vivek Rajagopal, Ian J. Sarembock, Andreas Brieke, Gilbert H.L. Tang, Yanru Li, Maria C. Alu, JoAnn Lindenfeld, William T. Abraham, Samin K. Sharma, Michael J. Mack, Gregg W. Stone

**Affiliations:** a The Zena and Michael A. Wiener Cardiovascular Institute, Icahn School of Medicine at Mount Sinai, New York, New York; b MedStar Health Research Institute, Washington, DC; c Department of Cardiology, Department of Internal Medicine, Baylor Scott & White The Heart Hospital – Plano, Plano, Texas; d Cardiovascular Institute of Los Robles Regional Medical Center, Thousand Oaks, California; e Structural Heart Program, Bakersfield Heart Hospital, Bakersfield, California; f Division of Cardiology, University of Virginia, Charlottesville, Virginia; g Department of Cardiology, Kaiser Permanente-San Francisco Hospital, San Francisco, California; h Department of Cardiology, Intermountain Medical Center, Murray, Utah; i Sanger Heart and Vascular Institute, Carolinas Medical Center, Charlotte, North Carolina; j Department of Cardiovascular Medicine, Heart and Vascular Institute, Cleveland Clinic, Cleveland, Ohio; k Piedmont Hospital, Atlanta, Georgia; l The Heart & Vascular Institute, The Christ Hospital and Lindner Clinical Research Center, Cincinnati, Ohio; m Heart failure Clinic, University Of Colorado Hospital, Aurora, Colorado; n Clinical Trials Center, Cardiovascular Research Foundation, New York, New York; o Advanced Heart Failure and Cardiac Transplantation Section, Vanderbilt Heart and Vascular Institute, Nashville, Tennessee; p Division of Cardiovascular Medicine, The Ohio State University College of Medicine, Columbus, Ohio

**Keywords:** GDMT, guideline-directed medical therapy, left ventricular ejection fraction, secondary mitral regurgitation, TEER, transcatheter edge-to-edge repair

## Abstract

**Background:**

Transcatheter edge-to-edge repair (TEER) improved outcomes in patients with heart failure (HF) and severe secondary mitral regurgitation (SMR) compared with guideline-directed medical therapy (GDMT) alone regardless of the severity of baseline left ventricular ejection fraction (LVEF). The study aimed to evaluate the effect of early changes in LVEF after TEER and GDMT alone in patients with HF and severe SMR.

**Methods:**

Within the COAPT trial, we evaluated outcomes according to changes in LVEF from baseline to 30 days. The primary outcome was all-cause death or HF hospitalization (HFH) between 30 days and 2 years.

**Results:**

Among 432 patients with paired echocardiographic data, 182 (42.1%) had increased LVEF (LVEF change 6.0% ± 4.9%) and 250 (57.9%) had a decrease or no change in LVEF (LVEF change –6.6% ± 5.6%) from baseline to 30 days. LVEF at 30 days increased more frequently with GDMT alone compared with TEER plus GDMT (51.4% vs 33.0%; *P* = .0001). Between 30 days and 2 years, there were no significant differences in death or HFH in the increase LVEF and the decrease LVEF groups (58.8% vs 51.4%; multivariable-adjusted HR, 0.97; 95% CI, 0.87-1.08; *P* = .59). TEER plus GDMT reduced the 30-day to 2-year rate of death or HFH compared with GDMT alone consistently in patients with increase LVEF and decrease LVEF (P_int_ = 0.75).

**Conclusions:**

Among patients with HF and severe SMR, early improvements in LVEF were more frequent with GDMT alone compared with TEER plus GDMT but were not associated with subsequent outcomes at 2 years. TEER reduced death or HFH during 2-year follow-up irrespective of early LVEF changes.

## Introduction

Secondary mitral regurgitation (SMR) in patients with heart failure (HF) is associated with a poor prognosis.^1^, ^2^, ^3^, ^4^ In the Cardiovascular Outcomes Assessment of the MitraClip Percutaneous Therapy for Heart Failure Patients With Functional Mitral Regurgitation (COAPT) trial, transcatheter edge-to-edge repair (TEER) with the MitraClip device (Abbott) reduced the rate of HF-related hospitalizations (HFH) and improved survival compared with guideline-directed medical therapy (GDMT) alone in patients with HF and moderate-to-severe or severe SMR who remained symptomatic despite maximally tolerated GDMT.^5^ As we previously reported, TEER was consistently effective in improving survival, rehospitalizations, and health status across the range of baseline left ventricular ejection fractions (LVEF) enrolled in the trial.^6^^,^^7^

Chronic mitral regurgitation (MR) is associated with LV volume overload and maladaptive remodeling over time. In patients with HF and LV dysfunction, SMR increases LV wall stress and promotes further LV dilatation.^8^ Surgical correction of MR increases afterload which may lead to an early postoperative decline in LVEF.^9^, ^10^, ^11^, ^12^, ^13^, ^14^ However, most of these analyses have been in the context of mitral valve (MV) surgery and were limited to patients with primary MR; the changes in LVEF after nonsurgical correction of SMR in HF have not been reported. Whether early improvements in LVEF after correction of MR provide prognostic information is also unknown.^9^, ^10^, ^11^, ^12^, ^13^ We, therefore, sought to investigate the association between early changes in LVEF and 2-year outcomes after TEER in patients with SMR and HF in this post hoc analysis from the COAPT trial.

## Methods

### Study design

The COAPT trial was an international, open-label, multicenter, randomized trial that evaluated TEER with the MitraClip device in symptomatic patients with HF and SMR. The COAPT trial design and principal results have been reported.^5^ In brief, eligible patients had ischemic or nonischemic cardiomyopathy with a site-assessed LVEF of 20%-50% and LV end-systolic diameter <7 cm. Moderate-to-severe (grade 3+) or severe (grade 4+) SMR was required in all patients and was confirmed at an echocardiographic core laboratory before enrollment, and all patients remained symptomatic despite the use of maximally tolerated GDMT.^15^ A central eligibility committee confirmed that the patient met all the enrollment criteria and categorized the patient’s expected risk of surgery-related complications and mortality. The institutional review board at each participating site approved the study, and all patients provided informed, written consent. The data supporting this study for further substudies may be made available upon request to the corresponding author from qualified investigators, contingent upon approval from the COAPT publications committee.

Enrolled patients were randomly assigned in a 1:1 ratio to TEER plus GDMT or to GDMT alone. Clinical follow-up was performed at 1, 6, 12, 18, and 24 months and is ongoing annually thereafter through 5 years. Echocardiographic follow-up was performed at 30 days, 6 months, 1 year, and 2 years for all randomized participants. Periodic assessments also included 6-minute walk distance, and quality-of-life measures including the New York Heart Association (NYHA) functional class and the Kansas City Cardiomyopathy Questionnaire score at baseline and at 1, 6, 12, 18, and 24 months after randomization. At the 2-year follow-up visit, patients in the control arm who still met all enrollment criteria were allowed to crossover and be treated with TEER. The present report thus truncates analysis at 2 years to preserve the intention-to-treat analysis.

For the present study, patients were categorized as those who had an early increase in LVEF, defined as an LVEF change from baseline to 30 days of >0% (the increase LVEF group), and those who had no change or a decrease in LVEF from baseline to 30 days (the decrease LVEF group). All transthoracic echocardiograms were evaluated by an independent echocardiographic core laboratory (Medstar Health Research Institute).

### End points

The primary outcome of interest for the present analysis was the composite of all-cause death or HFH between 30 days and 2 years after randomization. Secondary outcomes included all-cause death, cardiovascular death, HF-related death, all hospitalizations, cardiovascular hospitalizations, HFH, and echocardiographic assessments during follow-up. Adverse events were adjudicated by an independent events committee with the use of original source documents.

### Statistical analysis

Categorical variables were compared with the Fisher exact test or the χ^2^ test. Continuous variables were compared with *t* tests for normally distributed data or the Wilcoxon rank-sum test for nonnormally distributed data. Analysis of covariance was used to compare mean changes in continuous outcome measures from baseline to follow-up between groups. Multivariable linear regression analysis was performed to determine from the covariates in Table 1 and the randomized treatment group the independent predictors of a change in LVEF as a continuous variable from baseline to 30 days. Event rates were based on Kaplan-Meier estimates in time-to-first-event analyses and were compared with the log-rank test. Because the proportionality assumption was not met, the independent association between changes in LVEF between baseline and 30 days and outcomes from 30 days through 2 years was evaluated in multivariable logistic regression models with an adjustment for time-to-event (the logarithm of the time-to-event was included as an offset term). Logistic regression models were adjusted for baseline LVEF, randomized treatment to TEER vs GDMT, age, sex, ischemic (vs nonischemic) cardiomyopathy, NYHA class IV (vs ≤III), brain natriuretic peptide, chronic obstructive lung disease, creatinine clearance, anemia, previous percutaneous coronary intervention, HFH within the prior year, MR 4+ (vs 3+), and right ventricular systolic pressure. Multiple imputation was used to account for missing covariate data. The significance of the differences in the treatment effect of TEER plus GDMT vs GDMT alone according to the changes in LVEF between baseline and 30 days was assessed in Cox regression models for the full trial population, including main effect terms (eg, change in LVEF and assigned treatment) and interaction terms (eg, change in LVEF × assigned treatment) for each outcome of interest. The relationship between the changes in LVEF from baseline to 30 days as a continuous variable and clinical outcomes between 30 days and 2 years was also modeled by penalized smoothing spline analysis with 2 degrees of freedom and no specified knots. A 2-sided *P* < .05 was considered to indicate statistical significance. All statistical analyses were performed with SAS version 9.4 (SAS Institute).

**Table 1:** Baseline clinical and echocardiographic characteristics of patients with an increase vs decrease or no change in left ventricular ejection fraction from baseline to 30 days.

	iLVEF (n = 182)	dLVEF (n = 250)	*P* value
Age, y	72.9 ± 10.7	70.6 ± 11.8	.04
Male sex	64.3% (117/182)	65.2% (163/250)	.84
Diabetes mellitus	32.4% (59/182)	37.2% (93/250)	.30
Hypertension	80.2% (146/182)	76.8% (192/250)	.39
Hypercholesterolemia	50.5% (92/182)	54.4% (136/250)	.43
Previous myocardial infarction	50.0% (91/182)	52.0% (130/250)	.68
Previous percutaneous coronary intervention	45.6% (83/182)	44.8% (112/250)	.87
Previous stroke or transient ischemic attack	15.9% (29/182)	17.2% (43/250)	.73
Peripheral vascular disease	17.0% (31/182)	16.0% (40/250)	.77
Chronic obstructive lung disease	19.2% (35/182)	22.4% (56/250)	.43
History of atrial fibrillation or flutter	53.3% (97/182)	56.0% (140/250)	.58
Body mass index, kg/m^2^	26.9 ± 6.2	27.0 ± 5.5	.82
Creatinine clearance, mL/min	49.1 ± 26.1	52.2 ± 28.0	.26
Anemia	28.6% (52/182)	19.6% (49/250)	.03
Ischemic cardiomyopathy	58.8% (107/182)	60.4% (151/250)	.74
NYHA class III or IV	61.0% (111/182)	60.6% (151/249)	.94
Heart failure hospitalization within the prior year	59.3% (108/182)	55.2% (138/250)	.39
Previous cardiac resynchronization therapy	35.7% (65/182)	38.0% (95/250)	.63
Previous defibrillator implant	61.0% (111/182)	68.8% (172/250)	.09
B-type natriuretic peptide level, pg/mL	1097.9 ± 1037.9	961.6 ± 1005.4	.26
N-terminal pro–B-type natriuretic peptide level, pg/mL	4950.02 ± 5098.61	4599.1 ± 5815.9	.73
KCCQ-OS score	53.5 ± 22.7	52.6 ± 22.7	.69
6MWD, m	245.0 ± 124.8	252.6 ± 126.9	.54
Echocardiographic characteristics (core laboratory)			
Mitral regurgitation severity 4+	47.3% (86/182)	50.4% (126/250)	.52
Left ventricular end-systolic diameter, cm	5.3 ± 0.8	5.3 ± 0.9	1.00
Left ventricular end-diastolic diameter, cm	6.2 ± 0.7	6.0 ± 0.7	.40
Left ventricular ejection fraction, %	27.9 ± 8.7	33.0 ± 9.2	<.0001
Total stroke volume, mL	51.0 ± 20.1	63.1 ± 21.6	<.0001
Pulmonary artery diameter, cm	2.8 ± 0.4	2.7 ± 0.4	.04
Right ventricular systolic pressure, mm Hg	45.3 ± 13.5	43.6 ± 13.5	.24
Tricuspid regurgitation severity ≥3+	0.6% (1/177)	0.8% (2/247)	.77

Continuous data are presented as mean ± SD. Categorical data are presented as % (n/N).

6MWD, 6-minute walk distance; dLVEF, decreased (or unchanged) left ventricular ejection fraction; iLVEF, increased left ventricular ejection fraction; KCCQ-OS, Kansas City Cardiomyopathy Questionnaire Overall Summary Score; NYHA, New York Heart Association.

## Results

### Patients and change in LVEF

From December 2012 through June 2017, 614 patients at 78 centers in the United States and Canada were enrolled in the COAPT trial. Baseline and 30-day follow-up echocardiograms suitable for LVEF analysis by the echocardiographic core laboratory were available in 432 patients (including 214 and 218 randomized to GDMT alone and TEER plus GDMT respectively), representing the current analytic population. The mean LVEF in the entire cohort was 30.8% ± 9.3% at baseline and 29.6% ± 9.9% at 30 days (*P* = .001). There were 182 (42.1%) patients in the increase LVEF group (mean ΔLVEF from baseline to 30 days 6.0% ± 4.9%, range 0.4% to 24.0%) and 250 (57.9%) patients in the decrease LVEF group (mean ΔLVEF –6.6% ± 5.6%, range –27.0% to 0.0%). Patients in the increase LVEF group had lower baseline LVEF than those in the decrease LVEF group (27.9% ± 8.7% vs 33.0% ± 9.2%; *P* < .0001) as well as lower LV stroke volume (Table 1). There were no other significant differences in the baseline characteristics between the 2 groups. Patients who were excluded from this analysis due to missing echocardiograms at either baseline or 30 days were slightly older than the analysis population, with a slightly higher prevalence of hypertension and lower creatinine clearance at baseline, but were otherwise similar to the analyzed population. Importantly, the proportion of missingness did not differ by randomized treatment arm (Supplemental Table S1).

The distribution of the early changes in LVEF in the randomized groups is shown in Supplemental Figure S1. The mean change in LVEF from baseline to 30 days was 0.4% ± 7.4% in the GDMT alone group and –2.9% ± 8.6% in the TEER plus GDMT group (*P* < .001) (see also Supplemental Figure S2). More patients randomized to GDMT alone compared with TEER plus GDMT had increased LVEF (51.4% vs 33.0%; *P* < .0001). By multivariable analysis, the independent predictors of a decline in LVEF as a continuous measure from baseline to 30 days were randomization to TEER plus GDMT, diabetes, hypertension, lower body mass index, anemia, and higher LVEF at baseline (Table 2).

**Table 2:** Multivariable predictors of change in left ventricular ejection fraction from baseline to 30 days.

Covariate	Estimate (95% CI)	*P* value
Randomized treatment (MitraClip vs GDMT)	–3.78 (–6.13, –1.43)	.002
Age (per 5 y)	–0.11 (–0.79, 0.58)	.76
Sex (male vs female)	2.46 (–0.54, 5.46)	.11
Diabetes mellitus	–4.49 (–7.28, –1.70)	.002
Hypertension	4.07 (0.82, 7.33)	.016
Hypercholesterolemia	–1.54 (–3.96, 0.89)	.22
Previous myocardial infarction	1.94 (–1.12, 5.01)	.22
Previous percutaneous coronary intervention	–1.70 (–4.67, 1.26)	.26
Previous stroke or transient ischemic attack	1.28 (–2.05, 4.61)	.45
Peripheral vascular disease	3.06 (–0.08, 6.20)	.059
Chronic obstructive lung disease	–0.23 (–3.23, 2.77)	.88
History of atrial fibrillation or flutter	–0.41 (–2.99, 2.18)	.76
Body mass index (per 5 kg/m^2^)	1.30 (0.07, 2.53)	.041
Creatinine clearance (per 5 mL/min)	–0.17 (–0.49, 0.15)	.30
Anemia	2.97 (0.05, 5.89)	.049
Ischemic cardiomyopathy	–0.25 (–3.92, 3.43)	.90
NYHA class III or IV (vs I or II)	2.34 (–0.68, 5.36)	.13
Heart failure hospitalization within the prior year	0.33 (–2.40, 3.06)	.81
Resynchronization (CRT-D or CRT-P)	–2.68 (–5.58, 0.22)	.07
Defibrillator (ICD or CRT-D)	–1.59 (–4.90, 1.72)	.35
B-type natriuretic peptide level (per 5 pg/mL)	–0.00 (–0.01, 0.01)	.60
KCCQ-OS (per 5 points)	–0.03 (–0.36, 0.31)	.88
6MWD (per 50 m)	0.50 (–0.10, 1.10)	.11
Mitral regurgitation severity 4+ (vs 3+)	2.27 (–0.31, 4.85)	.09
Left ventricular end-systolic diameter (cm)	–2.70 (–6.45, 1.06)	.16
Left ventricular end-diastolic diameter (cm)	1.48 (–2.75, 5.72)	.49
Left ventricular ejection fraction (%)	–0.37 (–0.61, –0.14)	.002
Total stroke volume (per 5 mL)	–0.42 (–0.85, 0.01)	.057
Pulmonary artery diameter (cm)	–1.57 (–4.71, 1.57)	.33
Right ventricular systolic pressure (per 5 mm Hg)	–0.01 (–0.52, 0.51)	.98

6MWD, 6-minute walk distance; CRT, cardiac resynchronization therapy; GDMT, guideline-directed medical therapy; ICD, implantable cardiac defibrillator; KCCQ-OS, Kansas City Cardiomyopathy Questionnaire Overall Summary Score; NYHA, New York Heart Association.

### Outcomes according to change in LV function

**Figure 1:**
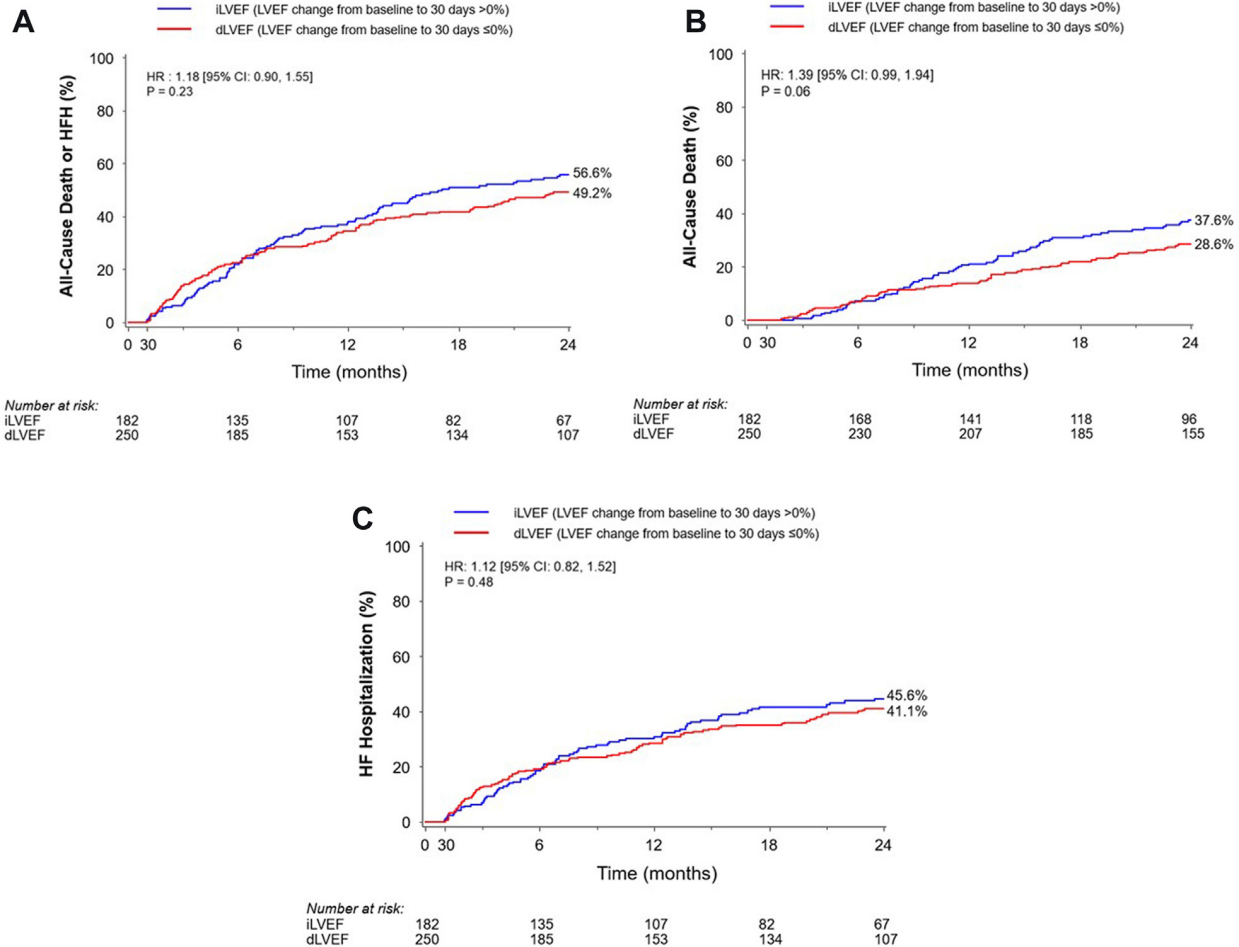
**Kaplan-Meier time-to-first event curves for outcomes between 30 days and 2 years according to changes in left ventricular ejection fraction (LVEF) from baseline to 30 days.** (**A**) All-cause death or heart failure hospitalization (HFH). (**B**) All-cause death. (**C**) HFH. dLVEF, decreased (or unchanged) left ventricular ejection fraction; HR, hazards ratio; iLVEF, increased left ventricular ejection fraction.

**Figure 2:**
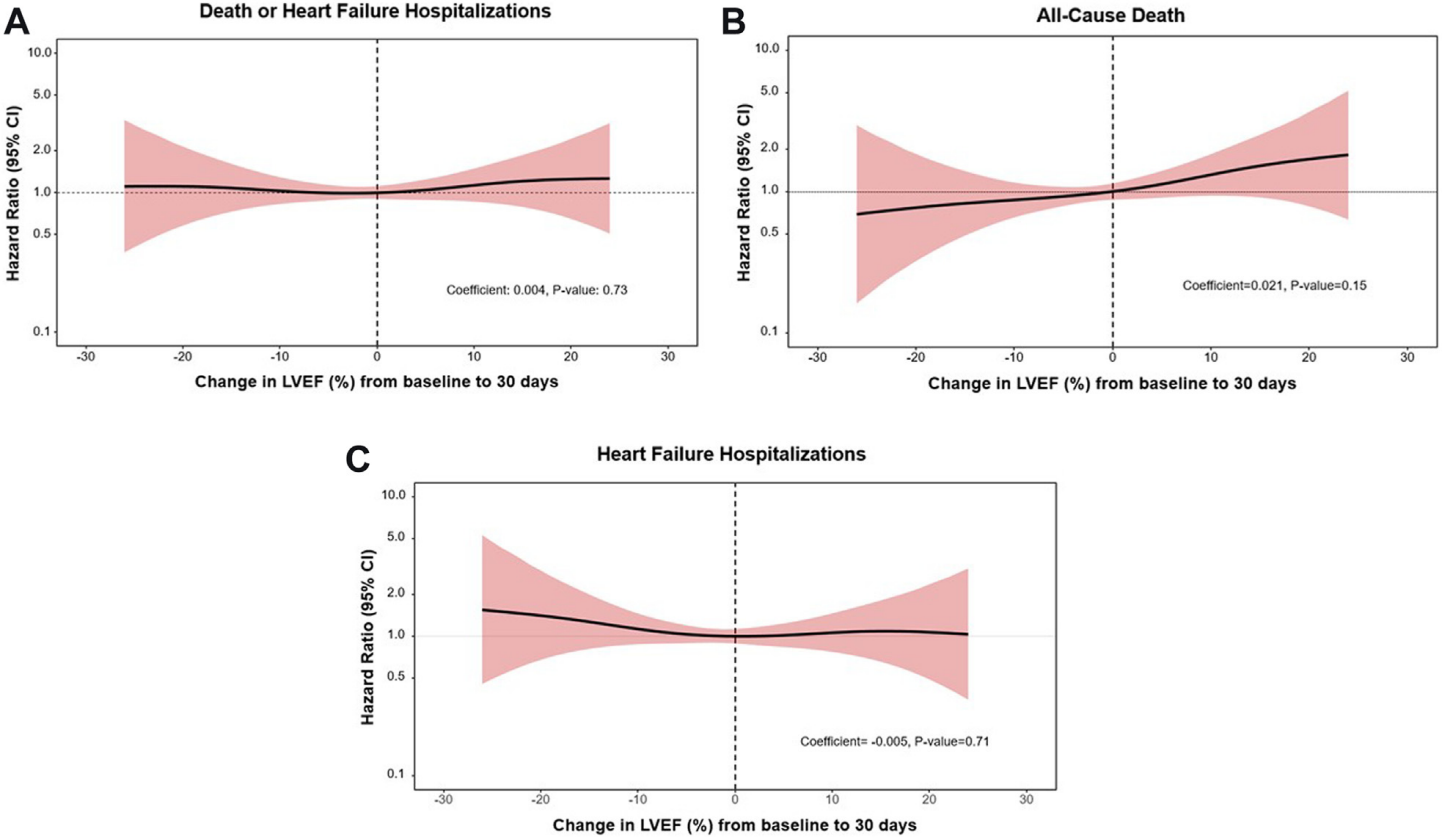
**Cubic spline regression for outcomes between 30 days and 2 years according to changes in left ventricular ejection fraction (LVEF) from baseline to 30 days.** (**A**) All-cause death or heart failure hospitalization (HFH). (**B**) All-cause death. (**C**) HFH.

There were no significant differences in the composite rates of all-cause death or HFH, or all-cause death and HFH alone between 30 days to 2 years in patients with increased LVEF or decreased LVEF at 30 days (Figure 1). By multivariable analysis, there was no significant association between either the baseline LVEF or the change in LVEF from baseline to 30 days and clinical outcomes between 30 days and 2 years (Figure 2 and Table 3). Nor were there significant differences in echocardiographic parameters of LV remodeling or clinical outcomes between the increase LVEF and decrease LVEF groups in the randomized treatment groups separately (Supplemental Tables S2 and S3). As a sensitivity analysis, we compared patients with LVEF change >10% vs ≤10%. At 2 years, there were no significant differences in death or hospitalization for HF (59.0% vs 49.6% respectively; RR, 1.28; 95% CI, 0.96-1.71) or in total rehospitalizations (73.8% vs 71.1% respectively; RR, 1.01; 95% CI, 0.78-1.31) in this cohort. Finally, there were no significant differences in the Kansas City Cardiomyopathy Questionnaire between patients with an increased vs decreased in LVEF (63.2 ± 26.4 vs 66.0 ± 24.3; *P* = .37).

**Table 3:** Adjusted relationships between change in left ventricular ejection fraction from baseline to 30 days and cardiovascular outcomes between 30 days and 2 years.

	Odds ratio (95% CI)	*P* value
All-cause death or HFH	1.00 (0.86-1.16)	1.00
Cardiovascular death or HFH	0.98 (0.84-1.14)	.78
All-cause death	1.07 (0.91-1.25)	.42
Cardiovascular death	1.04 (0.88-1.24)	.62
All hospitalizations	0.90 (0.77-1.05)	.18
Cardiovascular hospitalizations	0.90 (0.77-1.04)	.15
HFH	0.90 (0.77-1.05)	.18

The following covariates were included in the multivariable model: change in LVEF between baseline and 30 days as a continuous variable per 1% increase, baseline LVEF, age, sex, ischemic vs nonischemic cardiomyopathy, chronic obstructive pulmonary disease, anemia, previous revascularization, previous heart failure-related hospitalization, New York Heart Association class, brain natriuretic peptide at baseline, creatine clearance at baseline, mitral regurgitation grade (3 or 4+), right ventricular systolic pressure. Missing variables were imputed; because the proportional hazard assumption was not met for the association between LVEF changes and outcomes, time-to-event outcomes were analyzed by logistic regression with an adjustment for time-to-event (the logarithm of the time-to-event was included as an offset term).

HFH, heart failure hospitalization.

### Long-term impact of randomized treatment according to early change in LVEF

**Figure 3:**
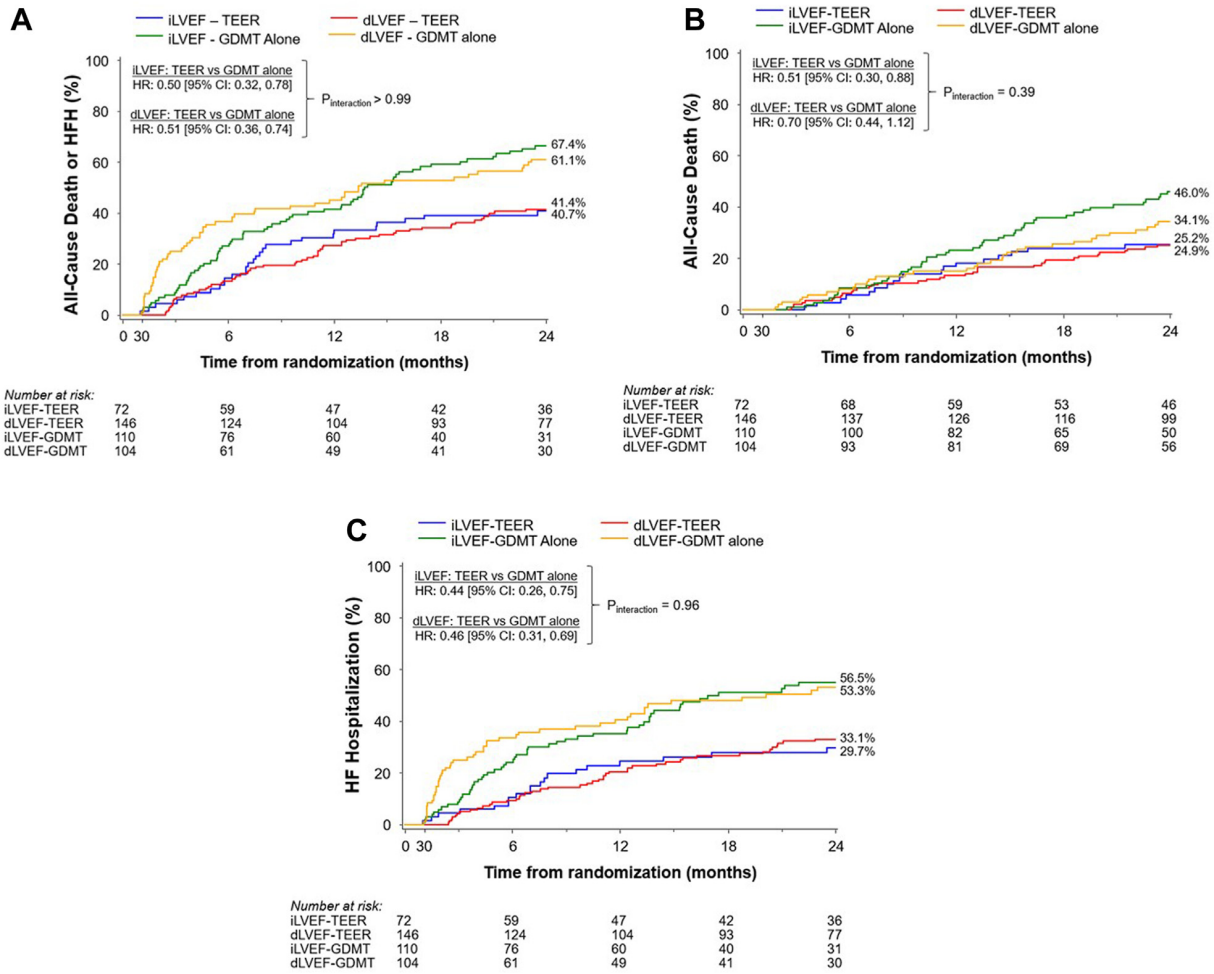
**Kaplan-Meier time-to-first event curves for outcomes between 30 days and 2 years according to changes in left ventricular ejection fraction (LVEF) from baseline to 30 days and randomized group.** (**A**) All-cause death or heart failure hospitalization (HFH). (**B**) All-cause death. (**C**) HFH. dLVEF, decreased (or unchanged) left ventricular ejection fraction; GDMT, guideline-directed medical therapy; HR, hazards ratio; iLVEF, increased left ventricular ejection fraction; TEER, transcatheter edge-to-edge repair.

Outcomes between 30 days and 2 years in the TEER plus GDMT vs GDMT alone groups according to the change in LVEF between baseline and 30 days are shown in Figure 3 and Central Illustration. TEER reduced all-cause death or HFH between 30 days and 2 years consistently in patients with increased LVEF (HR, 0.49; 95% CI, 0.31-0.76) and decreased LVEF (HR, 0.52; 95% CI, 0.39-0.70); P_int_ = 0.80. TEER also consistently reduced all-cause death (HR, 0.51; 95% CI, 0.30-0.88 and HR, 0.54; 95% CI, 0.38-0.78 respectively; P_int_ = 0.85) and HFH (HR, 0.44; 95% CI, 0.26-0.75 and HR, 0.49; 95% CI, 0.35-0.69 respectively; P_int_ = 0.75) between 30 days and 2 years in the increase LVEF and decrease LVEF groups.

**Central Illustration:**
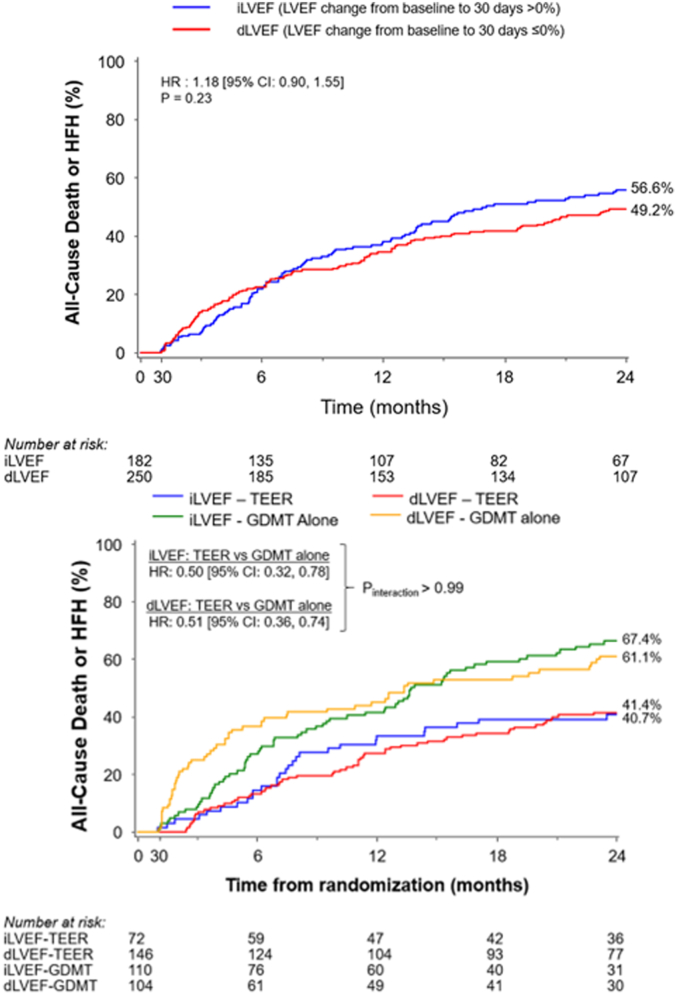
The benefits of transcatheter-edge-to-edge repair (TEER) in heart failure patients with severe secondary mitral regurgitation are realized independently of early changes in left ventricular ejection fraction (LVEF). dLVEF, decreased (or unchanged) left ventricular ejection fraction; GDMT, guideline-directed medical therapy; HFH, heart failure hospitalization; iLVEF, increased left ventricular ejection fraction.

## Discussion

In the present analysis from the COAPT trial, we examined the frequency of early changes in LVEF after treatment of SMR with TEER compared with GDMT alone and its association with clinical outcomes between 30 days and 2 years. The major findings of our study can be summarized as follows: (1) LVEF more frequently declined from baseline to 30 days after TEER compared with GDMT alone, and conversely more often improved after GDMT alone compared with TEER; (2) in addition to baseline LVEF, other independent predictors of change in LVEF from baseline to 30 days included diabetes, hypertension, anemia, body mass index, and baseline LVEF; (3) the change in LVEF from baseline to 30 days was not associated with the risk of adverse cardiovascular events between 30 days and 2 years; and (4) despite the greater reduction in early LVEF after TEER, treatment with the MitraClip device significantly and consistently reduced the composite rates of all-cause death or HFH and its components between 30-day and 2-year follow-up regardless of whether LVEF improved or declined at 30 days after randomization.

### Changes in LVEF after correction of secondary MR

Surgical correction of primary MR frequently results in a transient initial decline in LVEF in the postoperative period followed by progressive improvement and reverse LV remodeling.^9^, ^10^, ^11^, ^12^, ^13^ Persistent LV dysfunction following MV surgery for primary MR is more frequent among patients with baseline LV dysfunction and is associated with a poor prognosis.^9^, ^10^, ^11^, ^12^, ^13^ The current secondary analyses from the COAPT trial explored the frequency and clinical significance of early changes in LVEF among patients with HF and severe SMR who were treated using TEER plus GDMT vs GDMT alone. To the best of our knowledge, this is the first analysis investigating this issue in this high-risk patient cohort. In the entire population there tended to be a small reduction in LVEF from baseline through 30 days; despite a mean baseline LVEF of 30.8% ± 9.3% in the entire analytic cohort, approximately two-thirds of patients had some decrease in LVEF during this early period. Patients who were treated with TEER more frequently had a decline in LVEF compared with patients on GDMT alone (although even in the GDMT group nearly half of patients had an early decline in LVEF, reflecting progressive LV dysfunction in this high-risk cohort despite maximally tolerated GDMT). Consistent with prior observations in primary MR, correcting the MR may reduce LVEF due to the resultant increase in LV afterload (as well as a possible decrease in LV preload if diuresis is excessive after left atrial pressure declines from improving forward stroke volume). LVEF and stroke volumes were significantly lower at baseline in patients who had an increase in LVEF at 30 days in both the TEER + GDMT group and the GDMT alone group. Although this finding may in part reflect regression to the mean, it was not observed in previous primary MR studies. For example, among 861 patients with primary MR and preserved LVEF undergoing surgical MV repair or replacement, the decrease in postoperative LVEF was independently associated with a lower preoperative LVEF, as well as atrial fibrillation, advanced NYHA functional class, greater left ventricular end-diastolic and end-systolic dimensions, and larger left atrial size.^12^ It is possible that the hemodynamic effects of correcting SMR are different in patients with moderate or severe LV dysfunction at baseline compared with patients with primary MR and preserved LVEF.

### Association between LVEF changes and outcomes

In the present study, the early changes in LVEF were not associated with death or HFH between 30 days and 2 years, either in the GDMT alone group or the TEER plus GDMT group. This finding is again in contrast with observations in patients with primary MR with preserved LVEF undergoing MV surgery. In a large study of 1705 patients with severe primary MR and normal preoperative LVEF (>60%) undergoing surgical mitral repair, 314 (18.4%) had a decline in LVEF to <50% after surgery.^9^ This early LV impairment persisted in most patients during follow-up, with the LVEF recovering to preoperative levels (>60%) in only one-third of patients. A decrease in LVEF to <40% was associated with an increased risk of late death.^9^ The discrepant findings between this study and the present analysis may again be explained by differences in primary MR and SMR and baseline LV function. Primary MR is most commonly a disease of the MV leaflets or chordal apparatus, whereas SMR is most frequently caused by a dysfunctional, dilated LV with secondary mitral leaflet tethering and lack of coaptation. In SMR TEER leads to a reduction in mitral regurgitant volume and LV myocardial wall stretch (if not overcome by increased afterload). However, the extent of myocardial fibrosis may also determine the response to TEER independent of LVEF,^16^ and may have a prevailing effect on clinical outcomes. Regardless, despite the greater early reduction in LVEF after TEER plus GDMT compared with GDMT alone, TEER with the MitraClip device resulted in marked reductions in subsequent death and HFH consistently in patients in whom the LVEF either improved or fell within 30 days.

### Limitations

Entry criteria for COAPT required that patients with HF were symptomatic despite maximally tolerated GDMT, including prior treatment with cardiac resynchronization therapy or coronary revascularization as indicated.^17^ Therefore, the findings described in the present analysis cannot be generalized to asymptomatic patients or those not optimized on GDMT. COAPT also excluded end-stage HF patients and those with LVEF <20%, LV end-systolic dimension >7 cm, or severe pulmonary hypertension or right ventricular dysfunction. In such patients, the increase in afterload with MR correction may theoretically lead to an even greater early decline in LVEF than we observed, in some cases with clinical deterioration.^7^, ^8^, ^9^, ^10^, ^11^ To assess the impact of early changes in LVEF, we examined the effect that the ΔLVEF from baseline to 30 days had on outcomes between 30 days and 2 years. Although we did not note a deleterious effect of a reduction in LVEF in this early period on outcomes beyond 30 days, we cannot conclude that an early decline in LVEF was not related to outcomes within the first 30 days itself (although the overall rates of death or HFH within 30 days in the COAPT trial were relatively low^5^). Paired baseline and 30-day echocardiograms were not available in 182 of 614 enrolled subjects mostly due to patient withdrawal or loss-to-follow-up, or illness precluding traveling to the imaging center at 30 days. This imbalance may have introduced selection bias that may have affected our results. Finally, the hemodynamic and clinical effects observed in the present analysis are specific to TEER with the MitraClip device, which of note usually does not completely abolish MR. Future studies are required to characterize the ventricular and hemodynamic effects of other MV repair and replacement technologies that do completely eliminate MR. Although an independent core laboratory evaluated all transthoracic echocardiograms, post-TEER blinding was not possible. Finally, this was a nonprespecified analysis from the COAPT trial and it may have been underpowered to detect differences within the subgroups of patients with or without reduction in LVEF.

## Conclusions

In the COAPT trial, among patients with HF and moderate-to-severe or severe SMR and LV dysfunction, LVEF often declined within the first 30 days, more so after TEER treatment compared with GDMT alone. Although patients with worse baseline LVEF more frequently experienced an improvement in LVEF at 30 days, early LVEF reduction in LVEF was not associated with a greater risk of all-cause death or HFH between 30 days and 2 years. TEER with the MitraClip device significantly improved clinical outcomes compared with GDMT alone regardless of the change in LVEF from baseline to 30 days. The study suggests that among high-risk HF patients with severe SMR and the hemodynamic and myocardial profiles enrolled in COAPT, early changes in LVEF should neither be viewed optimistically (if improved) nor pessimistically (if reduced) and that the benefits of TEER in HF patients with severe SMR are realized independently of early changes in LVEF.
