# Supplementary material for: Changes in Left Ventricular Function and Outcomes After Trancatheter Edge-to-Edge Repair for Secondary Mitral Regurgitation
Source: J Soc Cardiovasc Angiogr Interv. 2024 Feb 23;3(5):101345. doi: 10.1016/j.jscai.2024.101345 (PMC11307461; doi:10.1016/j.jscai.2024.101345)

**APPENDIX**

**Supplemental Table S1. Baseline characteristics of patients excluded from the analysis population due to missing data compared with those included in the analysis**

**Supplemental Table S2.** **Echocardiographic characteristics during 2-year follow-up in patients with iLVEF and dLVEF from baseline to 30 days in patients treated with MitraClip plus GDMT or GDMT alone.**

**Supplemental Table S3. Clinical outcomes between 30 days and 2 years according to iLVEF and dLVEF from baseline to 30 days in patients treated with MitraClip plus GDMT or GDMT alone.**

**Supplemental Figure S1. Distribution in LVEF changes from baseline to 30 days in the MitraClip plus GDMT and GDMT alone groups.**

**Supplemental Figure S2. Box plot displaying the median and interquartile range and 95% confidence intervals for the changes in LVEF from baseline to 30 days in the MitraClip plus GDMT and GDMT alone groups.**

**Supplemental Table S1. Baseline characteristics of patients excluded from the analysis population due to missing data compared with those included in the analysis**

|  | **Excluded (n=182)** | **Included (n=432)** | **P value** |
| --- | --- | --- | --- |
| Randomized Treatment  MitraClip + GDMT  GDMT alone | 46.2%  53.8% | 50.5%  49.5% | 0.33 |
| Age (years) | 73.8 ± 10.5 | 71.6 ± 11.4 | 0.02 |
| Male sex | 62.1% | 64.8% | 0.52 |
| Diabetes mellitus | 42.3% | 35.2% | 0.10 |
| Hypertension | 85.7% | 78.2% | 0.03 |
| Hypercholesterolemia | 55.5% | 52.8% | 0.54 |
| Previous myocardial infarction | 52.2% | 51.2% | 0.81 |
| Previous percutaneous coronary intervention | 48.4% | 45.1% | 0.47 |
| Previous stroke or transient ischemic attack | 18.1% | 16.7% | 0.66 |
| Peripheral vascular disease | 20.9% | 16.4% | 0.19 |
| Chronic obstructive lung disease | 28.6% | 21.1% | 0.04 |
| History of atrial fibrillation or flutter | 56.0% | 54.9% | 0.79 |
| Body mass index (kg/m^2^) | 27.2 ± 6.1 | 27.0 ± 5.8 | 0.66 |
| Creatinine clearance (mL/min) | 45.5 ± 25.5 | 50.9 ± 27.2 | 0.03 |
| Anemia | 23.6% | 23.4% | 0.95 |
| Ischemic cardiomyopathy | 63.2% | 59.7% | 0.42 |
| NYHA class III or IV | 61.0% | 60.8% | 0.96 |
| Heart failure hospitalization within the prior year | 57.7% | 56.9% | 0.86 |
| Previous cardiac resynchronization therapy | 35.2% | 37.0% | 0.66 |
| KCCQ-OS score | 51.0 ± 23.9 | 53.0 ± 22.7 | 0.34 |

Continuous data are presented as mean ± standard deviation. Categorical data are presented as %. GDMT denotes guideline directed medical therapy; KCCQ-OS, Kansas City Cardiomyopathy Questionnaire Overall Summary Score; and NYHA, New York Heart Association.

**Supplemental Table S2.** **Echocardiographic characteristics during 2-year follow-up according to change in LVEF from baseline to 30 days in patients treated with MitraClip plus GDMT or GDMT alone.**

|  | **MitraClip plus GDMT** | | **p-value** | **GDMT alone** | | **p-value** |
| --- | --- | --- | --- | --- | --- | --- |
|  | **iLVEF (n=72)** | **dLVEF (n=110)** |  | **iLVEF (n=110)** | **dLVEF (n=184)** |  |
| **Baseline** |  |  |  |  |  |  |
| Mitral regurgitation severity 4+ (vs. 3+) | 45.8% (33/72) | 53.1% (111/209) | 0.29 | 48.2% (53/110) | 42.4% (78/184) | 0.33 |
| Left ventricular end-systolic diameter (cm) | 5.3 (0.7) | 5.3 (0.9) | 0.58 | 5.3 (0.9) | 5.3 (0.9) | 0.96 |
| Left ventricular end-diastolic diameter (cm) | 6.1 (0.6) | 6.2 (0.8) | 0.62 | 6.2 (0.8) | 6.2 (0.7) | 0.84 |
| Left ventricular end-systolic volume (mL) | 139.1 (58.3) | 134.2 (55.5) | 0.53 | 141.7 (67.2) | 129.4 (55.2) | 0.09 |
| Left ventricular end-diastolic volume (mL) | 188.7 (70.7) | 196.3 (68.7) | 0.42 | 193.5 (79.9) | 189.8 (68.5) | 0.67 |
| Left ventricular ejection fraction (%) | 27.2 (7.2) | 32.7 (9.2) | <0.0001 | 28.3 (9.6) | 33.1 (9.1) | <0.0001 |
| Total stroke volume (mL) | 49.7 (19.2) | 62.1 (23.0) | <0.0001 | 51.8 (20.8) | 60.4 (22.8) | 0.001 |
| Right ventricular systolic pressure (mmHg) | 43.2 (12.7) | 43.8 (13.0) | 0.79 | 46.5 (13.9) | 43.7 (13.7) | 0.11 |
| Tricuspid regurgitation severity ≥3+ (vs ≤2+) | 0% (0/70) | 0.5% (1/208) | 0.56 | 0.9% (1/107) | 1.7% (3/180) | 0.61 |
| **30 days** |  |  |  |  |  |  |
| Mitral regurgitation severity 4+ (vs. 3+) | 9.7% (7/72) | 5.9% (11/185) | 0.29 | 29.4% (32/109) | 26.7% (35/131) | 0.65 |
| Left ventricular end-systolic diameter (cm) | 5.2 (0.8) | 5.4 (0.9) | 0.054 | 7.2 (19.3) | 5.3 (0.8) | 0.27 |
| Left ventricular end-diastolic diameter (cm) | 6.0 (0.7) | 6.1 (0.8) | 0.21 | 9.1 (29.9) | 6.2 (0.7) | 0.27 |
| Left ventricular end-systolic volume (mL) | 112.1 (49.9) | 137.5 (52.2) | 0.0007 | 124.1 (59.5) | 128.6 (55.5) | 0.57 |
| Left ventricular end-diastolic volume (mL) | 166.7 (62.5) | 181.9 (60.0) | 0.08 | 183.0 (74.9) | 176.0 (62.1) | 0.46 |
| Left ventricular ejection fraction (%) | 33.8 (8.3) | 25.5 (8.1) | <0.0001 | 34.0 (10.4) | 27.8 (9.7) | <0.0001 |
| Total stroke volume (mL) | 54.5 (19.6) | 44.3 (16.5) | <0.0001 | 58.8 (23.8) | 47.4 (19.4) | 0.0002 |
| Right ventricular systolic pressure (mmHg) | 39.8 (11.8) | 39.8 (11.8) | 0.99 | 45.9 (15.5) | 41.7 (13.0) | 0.03 |
| Tricuspid regurgitation severity ≥3+ (vs ≤2+) | 9.7% (7/72) | 5.9% (11/185) | 0.29 | 0.9% (1/106) | 4.0% (5/126) | 0.15 |
| **2 years** |  |  |  |  |  |  |
| Mitral regurgitation severity 4+ (vs. 3+) | 2.6% (1/39) | 0% (0/113) | 0.09 | 28.3% (13/46) | 11.8% (8/68) | 0.03 |
| Left ventricular end-systolic diameter (cm) | 5.1 (1.0) | 5.3 (1.1) | 0.44 | 5.2 (1.0) | 5.3 (1.0) | 0.47 |
| Left ventricular end-diastolic diameter (cm) | 5.9 (0.7) | 6.1 (1.0) | 0.29 | 6.2 (0.9) | 6.2 (0.9) | 0.89 |
| Left ventricular end-systolic volume (mL) | 124.5 (53.7) | 129.4 (53.5) | 0.66 | 131.0 (51.6) | 137.5 (73.6) | 0.65 |
| Left ventricular end-diastolic volume (mL) | 179.8 (67.8) | 174.5 (59.3) | 0.69 | 187.0 (65.8) | 187.1 (80.1) | 0.99 |
| Left ventricular ejection fraction (%) | 31.1 (9.4) | 27.1 (9.0) | 0.04 | 30.5 (11.7) | 30.2 (12.7) | 0.92 |
| Total stroke volume (mL) | 55.3 (24.8) | 45.1 (16.3) | 0.01 | 56.1 (27.0) | 49.7 (18.9) | 0.19 |
| Right ventricular systolic pressure (mmHg) | 37.8 (12.8) | 40.9 (13.3) | 0.26 | 44.3 (17.3) | 36.8 (13.1) | 0.02 |
| Tricuspid regurgitation severity ≥3+ (vs ≤2+) | 3.6% (1/28) | 0% (0/79) | 0.09 | 3.7% (1/27) | 2.4% (1/41) | 0.76 |

Continuous data are reported as mean (standard deviation). GDMT = Guideline-Directed Medical Therapy; TIA = transient ischemic attack; LVAD = left ventricular assist device; dLVEF = decreased (or unchanged) left ventricular ejection fraction; iLVEF = increased left ventricular ejection fraction

**Supplemental Table S3. Clinical outcomes between 30 days and 2 years according to change in LVEF from baseline to 30 days in patients treated with MitraClip plus GDMT or GDMT alone.**

|  | **MitraClip** **plus GDMT** | | **p-value** | **GDMT alone** | | **p-value** |
| --- | --- | --- | --- | --- | --- | --- |
|  | **iLVEF (n=72)** | **dLVEF (n=110)** |  | **iLVEF (n=110)** | **dLVEF (n=184)** |  |
| Death or hospitalization for heart failure | 39.4% (27) | 42.2% (82) | 0.82 | 67.4% (69) | 64.1% (102) | 0.87 |
| Death from cardiovascular cause or hospitalization for heart failure | 36.3% (24) | 39.3% (74) | 0.79 | 62.7% (63) | 61.8% (97) | 0.92 |
| Death |  |  |  |  |  |  |
| All-cause | 25.2% (18) | 26.0% (51) | 0.93 | 46.0% (48) | 43.0% (72) | 0.81 |
| Cardiovascular | 17.9% (12) | 20.8% (39) | 0.63 | 38.9% (39) | 36.6% (59) | 0.87 |
| Related to heart failure | 8.2% (5) | 10.3% (18) | 0.61 | 26.7% (24) | 23.3% (35) | 0.81 |
| Not related to heart failure | 10.6% (7) | 11.8% (21) | 0.85 | 16.7% (15) | 17.5% (24) | 0.95 |
| Non-cardiovascular | 8.9% (6) | 6.5% (12) | 0.53 | 11.5% (9) | 10.1% (13) | 0.82 |
| Hospitalizations |  |  |  |  |  |  |
| All-cause | 63.0% (39) | 64.8% (114) | 0.85 | 79.5% (77) | 79.9% (114) | 0.48 |
| Cardiovascular | 40.4% (26) | 50.2% (91) | 0.35 | 64.0% (62) | 66.4% (98) | 0.40 |
| Related to heart failure | 29.7% (19) | 32.9% (59) | 0.79 | 56.5% (54) | 53.4% (79) | 0.85 |
| Not related to heart failure | 20.2% (13) | 28.6% (49) | 0.29 | 26.9% (23) | 34.4% (48) | 0.13 |
| Non-cardiovascular | 45.5% (28) | 42.4% (73) | 0.79 | 47.5% (43) | 49.3% (68) | 0.88 |
| Neurological events (stroke or TIA) | 4.3% (3) | 4.6% (8) | 0.95 | 7.1% (6) | 9.5% (13) | 0.50 |
| Stroke | 2.9% (2) | 4.1% (7) | 0.78 | 3.6% (3) | 9.0% (12) | 0.13 |
| TIA | 1.4% (1) | 1.1% (2) | 0.80 | 3.6% (3) | 0.6% (1) | 0.14 |
| Myocardial infarction | 2.9% (2) | 4.7% (8) | 0.61 | 8.2% (7) | 6.6% (8) | 0.57 |
| LVAD implantation or heart transplantation | 1.5% (1) | 4.4% (7) | 0.38 | 7.8% (6) | 8.9% (13) | 0.47 |
| LVAD implantation | 0% (0) | 3.7% (6) | 0.14 | 6.8% (5) | 4.7% (7) | 0.85 |
| Heart transplantation | 1.5% (1) | 0.7% (1) | 0.45 | 1.0% (1) | 5.9% (8) | 0.08 |

Event rates are Kaplan-Meier estimated rates (n events). GDMT = Guideline-Directed Medical Therapy; TIA = transient ischemic attack; LVAD = left ventricular assist device; dLVEF = decreased (or unchanged) left ventricular ejection fraction; iLVEF = increased left ventricular ejection fraction

**Supplemental Figure S1. Distribution in LVEF changes from baseline to 30 days in the MitraClip plus GDMT and GDMT alone groups.**

**
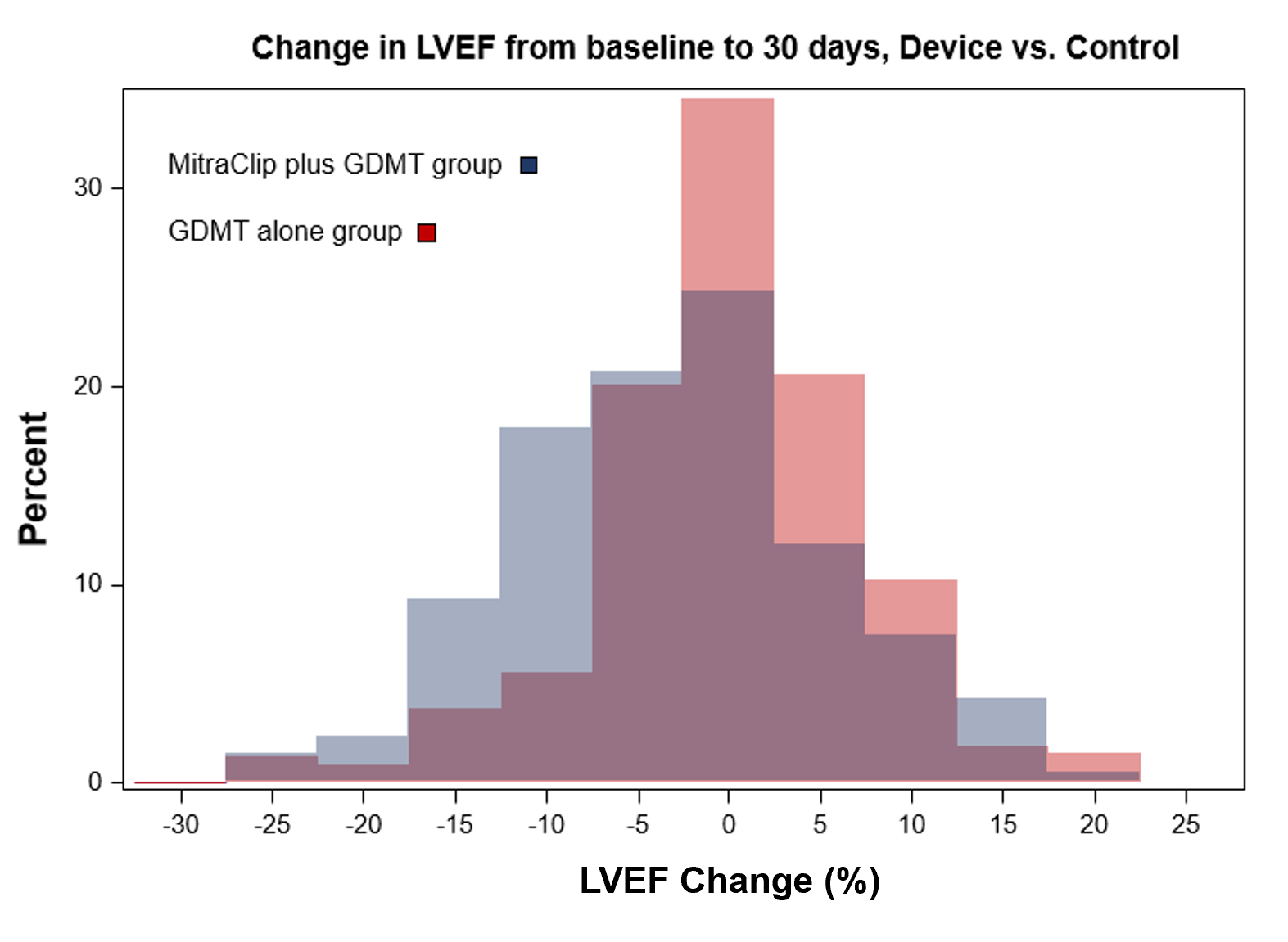
**

**Supplemental Figure S2. Box plot displaying the median and interquartile range and 95% confidence intervals for the changes in LVEF from baseline to 30 days in the MitraClip plus GDMT and GDMT alone groups.**


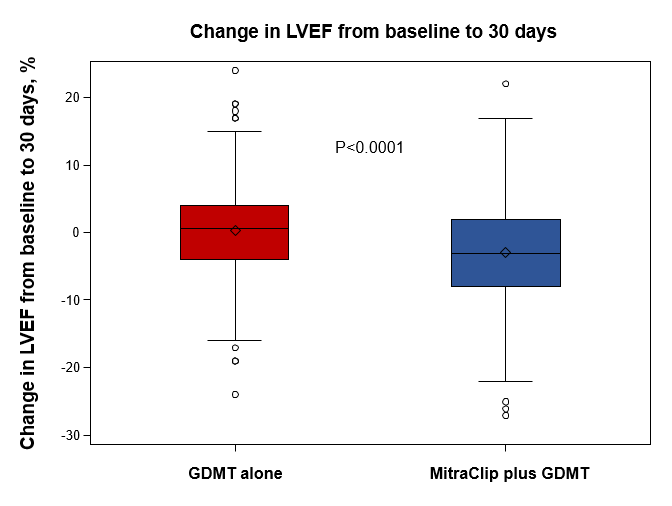

Supplement: Supplementary material [file mmc1.docx]
